# Chemical Analysis and Risk Assessment of Diethyl Phthalate in Alcoholic Beverages with Special Regard to Unrecorded Alcohol

**DOI:** 10.1371/journal.pone.0008127

**Published:** 2009-12-02

**Authors:** Jenny Leitz, Thomas Kuballa, Jürgen Rehm, Dirk W. Lachenmeier

**Affiliations:** 1 Chemisches und Veterinäruntersuchungsamt (CVUA) Karlsruhe, Karlsruhe, Germany; 2 Centre for Addiction and Mental Health (CAMH), Toronto, Canada; 3 Dalla Lana School of Public Health, University of Toronto, Toronto, Canada; 4 Institute for Clinical Psychology and Psychotherapy, TU Dresden, Dresden, Germany; East Carolina University, United States of America

## Abstract

**Background:**

Phthalates are synthetic compounds with a widespread field of applications. For example, they are used as plasticizers in PVC plastics and food packaging, or are added to personal care products. Diethyl phthalate (DEP) may be used to denature alcohol, e.g., for cosmetic purposes. Public health concerns of phthalates include carcinogenic, teratogenic, hepatotoxic and endocrine effects. The aim of this study was to develop and validate a method for determining phthalates in alcohol samples and to provide a risk assessment for consumers of such products.

**Methodology/Principal Findings:**

A liquid-liquid extraction procedure was optimized by varying the following parameters: type of extraction solvent (cyclohexane, *n*-hexane, 1,1,2-trichlorotrifluoroethane), the ratio extraction solvent/sample volume (1∶1 to 50∶1) and the number of extraction repetitions (1–10). The best extraction yield (99.9%) was achieved with the solvent 1,1,2-trichlorotrifluoroethane, an extraction solvent volume/sample volume ratio of 10∶1 and a double extraction. For quantification, gas chromatography/mass spectrometry with deuterated internal standards was used. The investigated samples were alcoholic beverages and unrecorded alcohol products from different countries (n = 257). Two unrecorded alcohol samples from Lithuania contained diethyl phthalate in concentrations of 608 mg/L and 210 mg/L.

**Conclusions/Significance:**

The consumption of the phthalate-positive unrecorded alcohols would exceed tolerable daily intakes as derived from animal experiments. Both positive samples were labelled as cosmetic alcohol, but had clearly been offered for human consumption. DEP seems to be unsuitable as a denaturing agent as it has no effect on the organoleptic properties of ethanol. In light of our results that DEP might be consumed by humans in unrecorded alcohols, the prohibition of its use as a denaturing agent should be considered.

## Introduction

Phthalic acid esters, commonly referred to as phthalates, are a group of industrial chemicals that have become ubiquitous environmental contaminants because of their widespread usage and high persistence in the environment [1]. They are generally colourless and odourless liquids with a low solubility in water [2]. Phthalates are used as plasticizers in many consumer products, such as household furnishings, in personal care products as vehicles for fragrance, in medical devices and children's toys, in food packaging, cleaning materials or insecticides. Since phthalates are not chemically bound to the plastic materials, they can leach out, migrate or evaporate into the air or into foodstuffs, for example. Accordingly, humans are exposed to phthalates through ingestion, inhalation and dermal contact throughout their lifetimes [3].

Another source of phthalates for human exposition is the use of diethyl phthalate (DEP) as a denaturing agent for ethyl alcohol. In Russia, alcohols denaturized by DEP have been available on the market for human consumption [4]. The risk of these surrogate alcohols, a subgroup of the category of unrecorded alcohol (see [5] for definition), often is not known to the consumers and it was hypothesized that these products currently lead to increased mortality in eastern European countries [4].

At the present time, a thorough toxicological evaluation is not available. Nevertheless, phthalates are suspected of causing health problems. The acute toxicity of phthalates is very low (LD_50_ 1–30 g/kg); however, the subchronic and chronic toxic effects of phthalates and their metabolites are of more importance. Toxicological investigations are now focused on carcinogenic, teratogenic and endocrine effects; some phthalates even show reproductive and developmental toxicity in animal experiments [2]. In general, the dose-response relationship of phthalates is difficult to evaluate. In Europe, there are tolerable daily intake (TDI) values only for some phthalates, such as di-2-ethylhexl phthalate (DEHP, 50 µg/kg bodyweight), di-n-butyl phthalate (DBP, 10 µg/kg bodyweight) or butylbenzyl phthalate (BBP, 500 µg/kg bodyweight) [6]–[8].

In the context of our ongoing investigation of unrecorded alcohol from different countries [9], [10], the aim of this study was to develop and validate a method for the determination of phthalates in alcoholic beverages. Especially the sample preparation should be as fast and inexpensive as possible, which is why we aimed to use a simple solvent extraction of the alcohols. Different parameters of this liquid-liquid extraction (LLE) method had to be optimized, including selection of the extraction solvent, the ratio extraction solvent volume/sample volume or the number of extraction repetitions. The results from our large collection of samples of unrecorded alcohols were used to provide a toxicological evaluation of these types of alcohols for consumers.

## Materials and Methods

### Chemicals and Reagents

Dimethyl phthalate (DMP), diethyl phthalate, diallyl phthalate (DAP), di-*iso*-butyl phthalate (DIBP), di-*n*-butyl phthalate (DBP), diethylhexyl adipate (DEHA), butyl-benzyl phthalate (BBP), di-2-ethylhexyl phthalate (DEHP), diheptyl phthalate (DHP) and di-*n*-octyl phthalate (DNOP) were all of GC grade and were purchased from Merck (Darmstadt, Germany). 3,4,5,6-*d_4_*-DEHP (*d_4_*-DEHP) was synthesized according to Loupy et al. [11] and Schwetlick [12]. The solvents 1,1,2-trichlorotrifluoroethane (>99.9%), ethyl acetate (>99.8%), cyclohexane (>99.9%) and *n*-hexane (>98.0%) were also purchased from Merck (Darmstadt, Germany).

A stock standard solution of the target analytes was prepared at a final concentration of about 1 g/L in cyclohexane/ethyl acetate (1∶1, v∶v). From this solution, a standard solution was prepared at a final concentration of 50 mg/L in cyclohexane/ethyl acetate (1∶1, v∶v). Calibration solutions of phthalates at nominal concentrations of 0.5, 1.0, 2.0, 4.0, 6.0, 10.0 and 20.0 mg/L were prepared by diluting the standard solution in cyclohexane/ethyl acetate (1∶1, v∶v). The nominal concentration of internal standard in each calibration dilution was about 7.5 mg/L.

### Glassware and Reagent Control

To avoid phthalate contamination, all glassware used in this study was rinsed with acetone and dried at 220°C at least 5 h. All glassware and reagents were checked for potential phthalate contamination. The solvents cyclohexane, ethyl acetate and *n*-hexane were checked by gas chromatographic-mass spectrometric (GC/MS) analysis once a week. 1,1,2-Trichlorotrifluoroethane was checked with every sample measurement.

### GC/MS Method

The GC/MS system used for analysis was a Trace GC in combination with a CTC Combi-PAL auto sampler and a Polaris Q mass spectrometer (Thermo Finnigan, Bremen, Germany). Data acquisition and analysis were performed using standard software supplied by the manufacturer (Xcalibur 1.3.1 and CTC Cycle Composer 1.5.3 for acquisition and Xcalibur 2.0.7 for data analysis). Substances were separated on a VF-Xms column (Factor Four, 29.3 m×0.25 mm I.D., film thickness 0.25 µm, Varian, Darmstadt, Germany). A 1 µL sample was injected into the split/splitless inlet in splitless mode (splitless for 1.5 min, split flow 10 mL/min) at 250°C. The temperature of the GC/MS transfer line was 280°C, the temperature of the ion source was 225°C. Helium with a constant flow rate of 1 mL/min was used as carrier gas. The oven temperature program was: 100°C, held for 1 min, 5°C/min up to 270°C, held for 0 min, 10°C/min up to 320°C, held for 10 min. The ion-trap mass spectrometer was operated in the electron ionization mode (70 eV) and the analytes were recorded in full-scan mode (*m/z* 40–300) (Table 1). For quantification, peak area ratios of the analytes to the internal standard *d_4_*-DEHP were calculated as a function of the concentration of substances.

**Table 1:** Retention time and selected ions for the analysis of the target phthalates.

Compound	Retention time (min)	Quantification ions(*m/z*)	Identification ions (*m/z*)
DMP	11.6	163	164
DEP	14.7	149	177
DAP	18.0	149	189, 132
DIBP	20.3	149	223, 150
DBP	22.2	149	150, 223
DEHA	29.5	129	147, 241
BBP	29.5	149	91, 206
DEHP	31.9	149	176, 279
DHP	32.0	149	265, 150
DNOP	34.9	149	150, 279
*d_4_*-DEHP	31.9	153	171

### Samples

A total of 257 samples submitted to the CVUA Karlsruhe were analyzed for the different phthalates listed in table 1. The samplings were conducted in the context of different international projects designed to characterize the quality of alcoholic beverages, including unrecorded alcohol. Further details on samples from Nigeria (illegally produced spirits; n = 6) [13], Mexico (tequila, mezcal; n = 24) [14], Lithuania (cheap spirits and cosmetic surrogate alcohols; n = 10) [5], Hungary (cheap fruit-derived spirits; n = 15) [5], Guatemala (surgarcane spirits (cuxa), commercial and clandestine variants; n = 22) [15], [16], Poland (commercial fruit wines and unrecorded spirits (moonshine); n = 44) [17], Vietnam (commercial and homemade spirits, mainly rice-based; n = 10) [18], and Brazil (commercial cachaça; n = 24) [19] were previously published. Furthermore, samples from India (spirits; n = 2), Ukraine (predominantly homeproduced spirits, so-called samogon; n = 61), Dominican Republic (unrecorded spirits; n = 2) and Romania (fruit spirits; n = 2), as well as samples legally available on the German market (spirits, mainly vodka; n = 35) were included in the study. The samplings were not representative but risk-oriented [20] as we have specifically searched for unrecorded products, more likely to be contaminated with diethyl phthalate from possible use of denatured alcohol (see references for details on sampling strategies in the respective countries).

### Sample Preparation

The sample preparation is a liquid-liquid extraction (LLE). 0.1 mL of the sample was placed in a glass test tube and 0.1 mL of internal standard (end concentration in the sample was about 7.5 mg/L) and 1 mL of 1,1,2-trichlorotrifluoroethane were added. The tube was closed with a ground-glass stopper and shaken on a Vortex mixer for 1 min. After centrifugation for 5 min (3000 rpm), the solvent phase (lower phase) was removed to a separate vial. A fresh 1 mL volume of 1,1,2-trichlorotrifluoroethane was added to the sample and the extraction was repeated. The two solvent phases were then combined and analyzed by GC/MS.

### Optimization and Validation Studies

Before validation, the LLE method had to be optimized by different parameters in order to completely separate the phthalates from the sample matrix. Three extraction solvents, cyclohexane, *n*-hexane and 1,1,2-trichlorotrifluoroethane, were compared by extracting 1 mL of an authentic alcoholic beverage sample with different volumes of these solvents. The ratio of extraction solvent volume/sample volume was chosen by extracting 0.1 mL of the same alcoholic beverage sample with different volumes (0.1–5 mL) of 1,1,2-trichlorotrifluoroethane. The optimum number of repetitions of the extraction procedure was determined by extracting 1 mL of the alcoholic beverage sample ten times successively with 1,1,2-trichlorotrifluoroethane.

For method validation and analytical quality assurance, we followed the demands for governmental food and alcohol control authorities [21]. Specifically, the principles outlined in ISO 17025 [22]. The method validation was conducted for DEP. For the validation, an authentic DEP-positive alcoholic beverage sample from Lithuania and a blank sample, i.e., a DEP-free vodka that was spiked with DEP-standard solution (end concentration about 25 mg/L), were extracted and analyzed several times daily (intraday, *n* = 6) and over several days (interday, *n* = 5) using the optimized procedure. The linearity of the calibration curves was evaluated between 0.1 and 20.0 mg/L. For the determination of the limit of detection (LOD) and the limit of quantitation (LOQ), a separate calibration curve in the range of LOD (0.1–1.0 mg/L) was established. The recovery rate was ascertained by adding DEP at two different concentrations (about 80 mg/L and 200 mg/L end concentration) to a blank sample (DEP-free vodka). The applicability of the procedure was proven by routine analysis of over 200 samples.

### Statistics

The experimental designs and calculations were done using the Software Package Design Expert v7 (Stat-Ease Inc., Minneapolis, MN, USA). The experiments were evaluated using Analysis of Variance (ANOVA) to find the significance of variables and their interactions in the models. The models were checked for consistency by looking at the lack of fit and possible outliers. Statistical significance was assumed at below the 0.05 probability level.

## Results

### Parameter Optimization for the LLE Method

**Figure 1:**
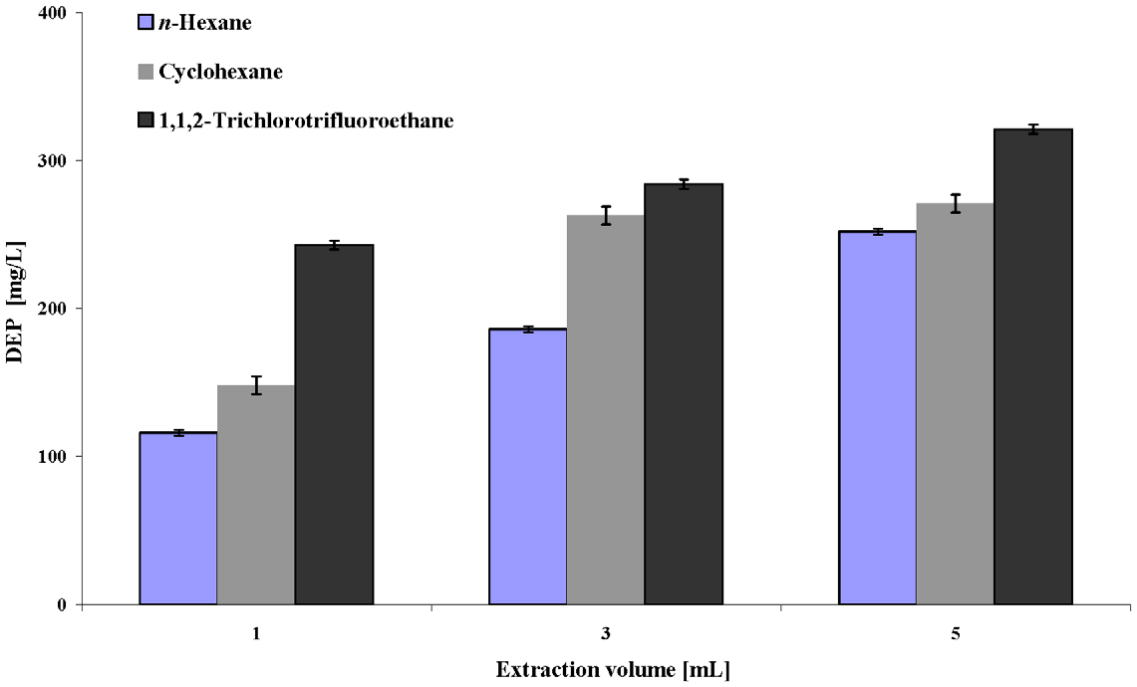
Comparison of the extraction of an authentic alcoholic beverage sample with three different extraction solvents.

In the literature, many different extraction solvents, such as cyclohexane, *n*-hexane, ethyl acetate or dichloromethane (each solvent also in addition with NaCl), have been suggested for the extraction of phthalates from various food matrices [23]–[27]. Another solvent, 1,1,2-trichlorotrifluoroethane, was suggested for the extraction of volatile compounds from alcoholic beverages [28]. Preliminary tests showed that of the mentioned solvents, cyclohexane, *n*-hexane and 1,1,2-trichlorotrifluoroethane, without the addition of NaCl, offered the best extraction results from alcoholic beverages. Figure 1 shows a comparison of these three solvents. The solvent type as well as solvent volume both significantly influence the extraction efficiency (ANOVA p<0.0001 for response surface model). 1,1,2-Trichlorotrifluoroethane extracted a significantly higher amount of phthalate from the sample than the two other solvents. Figure 1 also shows that a higher extraction solvent volume led to significantly higher amounts of extracted phthalate.

**Figure 2:**
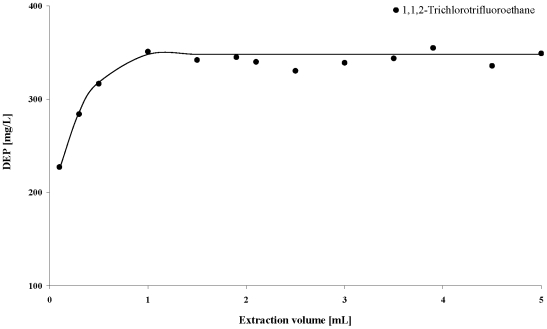
Evaluation of the optimal ratio extraction solvent volume/sample volume.

**Figure 3:**
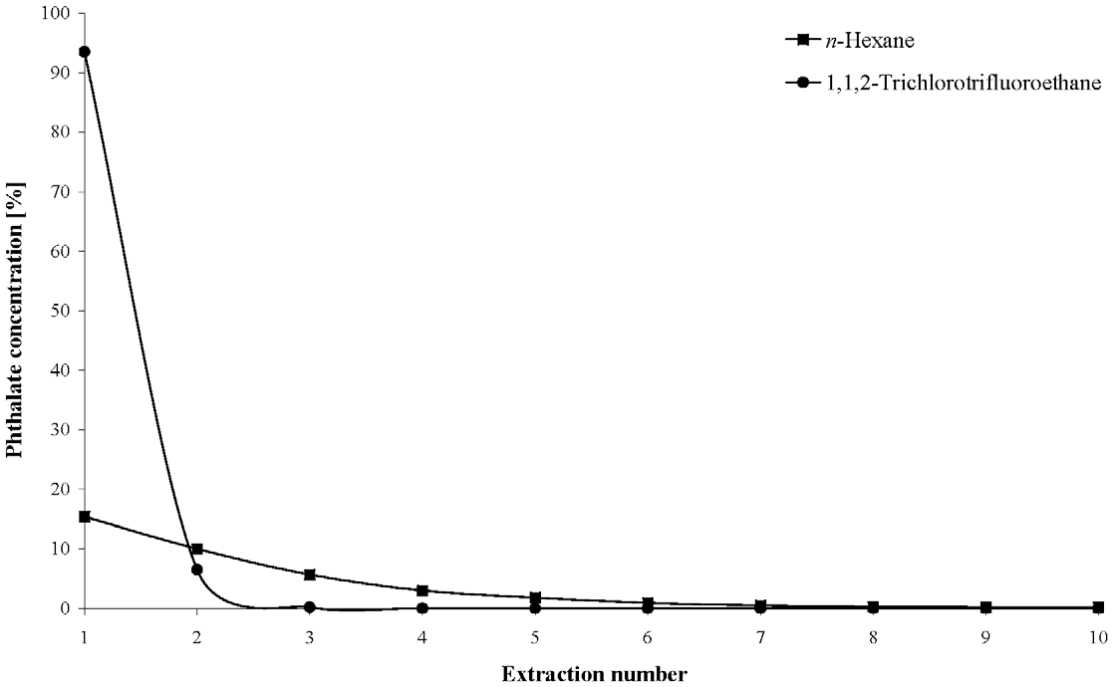
Repetition of the LLE and comparison of the two extraction solvents *n*-hexane and 1,1,2-trichlorotrifluoroethane.

For the following optimization experiments, 1,1,2-trichlorotrifluoroethane was chosen as the solvent for the LLE method. Figure 2 shows that the amount of extracted phthalate increases up to an extraction solvent volume of 1 mL, but exceeding that volume does not lead to any further significant increase in the extracted phthalate. Therefore, the optimal ratio extraction solvent volume/sample volume was 1 mL/0.1 mL. Repetition of the LLE showed that after two extractions, over 99.9% of the phthalate was extracted out of the sample (Figure 3). The optimal LLE parameters are summarized in Table 2. To improve precision and to correct for possible variability in extraction and GC/MS, deuterated DEHP was added as internal standard prior to the extraction in all further measurements.

**Table 2:** Results of LLE method optimization.

Parameter	Result
Extraction solvent	1,1,2-Trichlorotrifluoroethane
Ratio extraction solvent volume/sample volume	1 mL/0.1 mL
Repetition of LLE	1 (i.e. 2 extractions in total)

### Results of Method Validation and Sample Measurement

During routine analyses of 257 authentic alcoholic beverage samples, no interfering peaks from the spirit matrix were observed. Table 3 summarizes the method validation results for DEP. The assay was linear in the required concentration range between 0.1 and 20.0 mg/L with a regression coefficient of 0.9995. When determined according to DIN 32645 [29], the limit of detection (LOD) of DEP was 0.7 mg/L, the limit of quantitation (LOQ) was 2.6 mg/L. Both values were sufficient for our purposes. The precision expressed as relative standard deviation (RSD) of the optimized method for DEP never exceeded 9.0% (intraday) and 8.4% (interday) for the authentic alcoholic beverage sample and 8.2% (intraday) and 9.7% (interday) for the spiked sample. The method was verified by the recovery ranges of 103.9% (at 80 mg/L addition of DEP) and 110.4% (at 200 mg/L addition of DEP). The results of the method validation show that the method is precise and reproducible (Table 3).

**Table 3:** Results of method validation for DEP.

Parameter	Result
Linear range	0.1–20.0 mg/L
LOD^a^	0.7 mg/L
LOQ^a^	2.6 mg/L
Precision intraday^b^	9.0% (authentic sample); 8.2% (blank sample)
Precision interday^b^	8.4% (authentic sample); 9.7% (blank sample)
Recovery range	103.9% (at 80 mg/L); 110.4% (at 200 mg/L)

a Limit of detection (LOD) and quantitation (LOQ) were determined by establishing a separate calibration curve in the range of 0.1–1.0 mg/L. The limits were calculated from the residual standard deviation of the regression line [29].

b Precisions are expressed as relative standard deviation (RSD) (%), intraday (*n* = 6), interday (*n* = 5).

**Figure 4:**
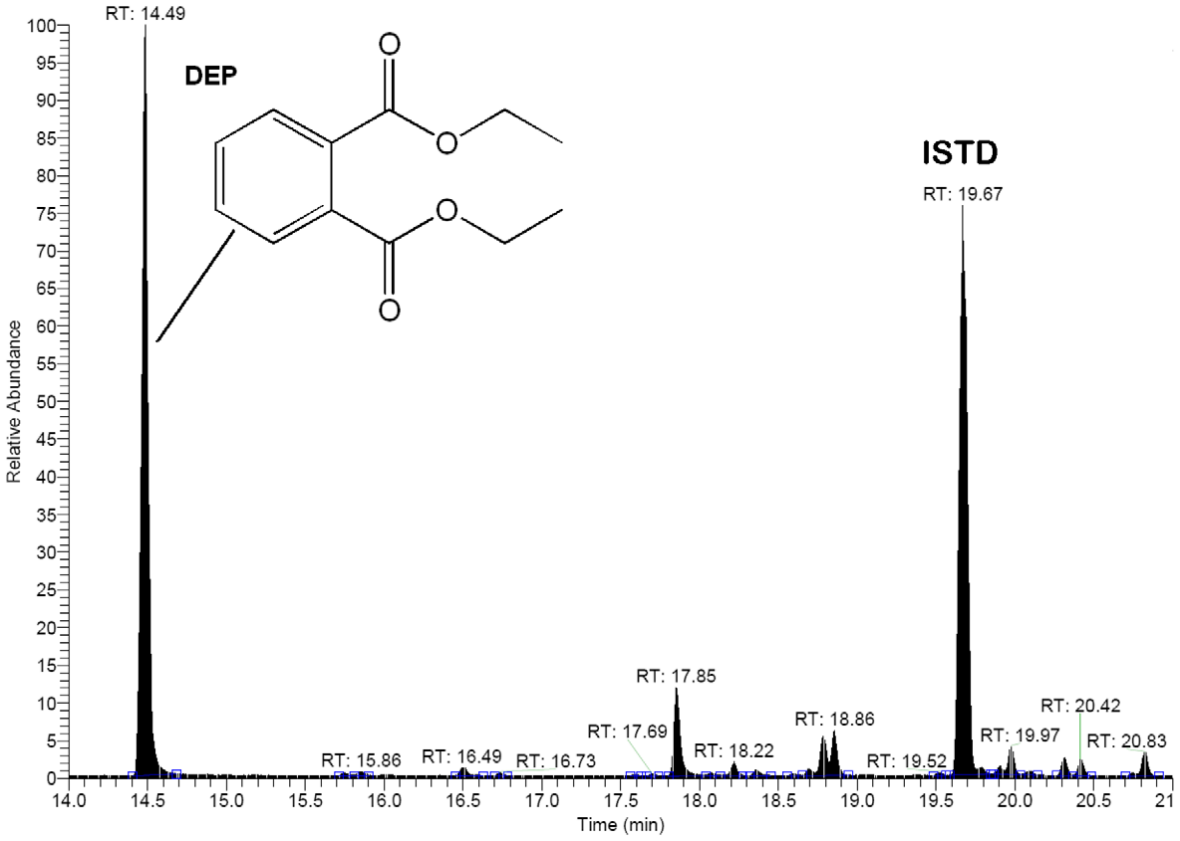
Chromatogram of an unrecorded alcohol sample from Lithuania containing 608 mg/L of diethyl phthalate (DEP); 3,4,5,6-*d_4_*-di-2-ethylhexyl phthalate (*d_4_*-DEHP) was used as internal standard (ISTD).

Only two of the 257 analysed authentic alcoholic beverage samples from different countries contained DEP, in concentrations of 608 mg/L and 210 mg/L (Figure 4). Both samples were obtained from Lithuania and were available on the market as cosmetic alcohol (“for Men Eau de Cologne” and “Cologne Syren Cupe Hb”). However, these were clearly sold for human consumption [5].

None of the other phthalates included in this study (DMP, DAP, DIBP, DBP, DEHA, BBP, DEHP, DHP, DNOP) was found in any of the 257 samples (this is also the reason why we have focused method optimization and validation purely on DEP).

## Discussion

The results of our large collective of samples suggest that the public health risk relevance of phthalates in alcohol is restricted to DEP in certain unrecorded alcohols. Recorded alcohols and migration from plastic bottles (many of the samples in the study were filled in plastic bottles) appear to be of negligible risk. The problem may also be restricted to legislations that allow the use of diethyl phthalate to denature alcohol. However, the sample size in some of the countries investigated was relatively small and does not allow a final conclusion on the worldwide scale of phthalate contamination in alcohol.

Regarding DEP, there are several reviews on its toxicological profile that raise concerns because of the ubiquitous occurrence. After inhalation, oral or dermal uptake, DEP is mainly hydrolyzed to ethanol and monoethyl phthalate, which is then excreted in the urine [30]. DEP exerts low acute toxicity; lethal doses are reported in the range of 9–31 g/kg [31]. No carcinogenic effects after dermal or oral exposure were detected in long-term studies with rodents [30], [32]. An oral reference dose (RfD) was set by the US Environmental Protection Agency [33] at 0.8 mg/kg bodyweight/day (48 mg/day for a 60-kg-human) with extrapolation from the short-term animal toxicity experiments of Brown et al. [34]. A working group of the WHO [35] estimated a TDI of 5 mg/kg bodyweight (300 mg/day for a 60-kg-human) from a NOAEL of 1600 mg/kg bodyweight for developmental effects, derived from the same study of Brown et al. [34]. In addition, phthalates were also suspected as being hepatotoxic, with a slightly higher NOAEL (3160 mg/kg/day for male rats [36]). According to the RfD and the TDI for DEP we estimated a drinking volume of the two DEP containing samples a 60-kg person could drink everyday without expecting adverse effects. Subsequently consumption of more than 79 mL–1.4 L of our positive samples could be critical (Table 4). Under assumption of a 60-kg person drinking 100 mL of phthalate contaminated alcohol with 40% vol, the alcohol could contain 480–3000 mg/L (120–750 g/hL of pure alcohol) of DEP to reach the RfD or TDI. While the use of DEP-containing products above this level might be safe for cosmetic use on the human skin [30], [37], we have major concerns about the oral use of these products. As the critical level in alcohol products appears to be in the upper mg/L-range, the detection limit of our procedure of 0.7 mg/L appears to be adequate for the purpose, and we have refrained from further sample preparation steps (e.g., solid phase extraction) that might improve the detection limit but also increase the time and cost of the assay.

**Table 4:** Toxicological evaluation of the DEP containing samples from Lithuania.

	Drinking volume for a 60-kg person to exhaust toxicological threshold	
Product	US EPA RfD (0.8 mg/kg bodyweight/day)	WHO TDI (5 mg/kg bodyweight/day)
Surrogate alcohol #1 (608 mg/L)	79 mL	493 mL
Surrogate alcohol #2 (210 mg/L)	229 mL	1429 mL

Further investigations are necessary to estimate the risk of DEP intake for humans. Some member states of the European Union still allow the use of DEP as a denaturing agent. In Germany, 0.5 kg DEP can be used for denaturing 100 L ethanol for the production of cosmetic agents or agents used to improve the odour [38]. In Russia, a regulation for the use of DEP as a denaturing agent for ethanol and ethanol-containing products existed [39]. However, this regulation appears to have been amended, without the inclusion of DEP [40].

The high amount of DEP in samples from Lithuania cannot be explained by the migration of DEP out of the packaging material. Instead, it documents the use of DEP as a denaturing agent for the alcohol or as an ingredient in the cosmetic as a vehicle for fragrance [37]. It can be assumed that this alcohol was only declared as perfume or eau-de-cologne to evade taxation, but in fact it was offered for human consumption.

Another aspect of the toxicological evaluation that cannot be excluded is the interaction of DEP with ethanol, which is also hepatotoxic. We also have found a third liver toxic compound, coumarin, in the same two products that contained DEP above international limits [5]. Therefore, we have three liver-toxic agents in the unrecorded alcohols (ethanol, DEP and coumarin). We currently do not have enough evidence to postulate a real public health threat, because the occurrence of DEP and coumarin in unrecorded alcohol is generally unknown. However, regions with high consumption of unrecorded alcohol also have higher incidence of liver cirrhosis, which cannot be explained by the volume and patterns of drinking alone [5]. Therefore, constituents in unrecorded alcohol, such as DEP, provide an interesting hypothesis to explain these increased effects, and are worthy of further investigation. The methodology developed in this study will be used to analyze a larger collective of unrecorded samples in the context of the Alcohol Measures for Public Health Research Alliance (AMPHORA), a collaborative project funded under the European Commission Seventh Framework Program.

Currently we can conclude that DEP seems to be unsuitable as a denaturing agent as it has no effect on the organoleptic properties of ethanol and can easily be separated by distillation [4]. For cosmetics, oils that are part of the recipe anyway or bitter agents such as denatonium benzoate (bitrex®) should be used as alternative denaturing agents [41]. Consequently, prohibition of the use of DEP as a denaturing agent should be considered, as its toxicological effects are still uncertain, but a clear potential public health risk exists.
